# There Is a Method to the Madness: Strategies to Study Host Complement Evasion by Lyme Disease and Relapsing Fever Spirochetes

**DOI:** 10.3389/fmicb.2017.00328

**Published:** 2017-03-02

**Authors:** Ashley L. Marcinkiewicz, Peter Kraiczy, Yi-Pin Lin

**Affiliations:** ^1^Division of Infectious Disease, Wadsworth Center, New York State Department of HealthAlbany, NY, USA; ^2^Institute of Medical Microbiology and Infection Control, University Hospital of Frankfurt am MainFrankfurt am Main, Germany

**Keywords:** Lyme disease, relapsing fever, spirochetes, *borrelia*, innate immunity, complement system, blood stream survival

## Abstract

Lyme disease and relapsing fever are caused by various *Borrelia* species. Lyme disease *borreliae*, the most common vector-borne pathogens in both the U.S. and Europe, are transmitted by *Ixodes* ticks and disseminate from the site of tick bites to tissues leading to erythema migrans skin rash, arthritis, carditis, and neuroborreliosis. Relapsing fever *borreliae*, carried by ticks and lice, trigger reoccurring fever episodes. Following transmission, spirochetes survive in the blood to induce bacteremia at the early stages of infection, which is thought to promote evasion of the host complement system. The complement system acts as an important innate immune defense mechanism in humans and vertebrates. Upon activation, the cleaved complement components form complexes on the pathogen surface to eventually promote bacteriolysis. The complement system is negatively modulated by a number of functionally diverse regulators to avoid tissue damage. To evade and inhibit the complement system, spirochetes are capable of binding complement components and regulators. Complement inhibition results in bacterial survival in serum (serum resistance) and is thought to promote bloodstream survival, which facilitates spirochete dissemination and disease manifestations. In this review, we discuss current methodologies to elucidate the mechanisms of *Borrelia* spp. that promote serum resistance and bloodstream survival, as well as novel methods to study factors responsible for bloodstream survival of Lyme disease *borreliae* that can be applied to relapsing fever *borreliae*. Understanding the mechanisms these pathogens utilize to evade the complement system will ultimately aid in the development of novel therapeutic strategies and disease prevention to improve human health.

## Complement evasion among lyme disease and relapsing fever spirochetes

The spirochete *Borrelia* is the bacterial agent causing both Lyme disease (LD) and relapsing fever (RF) (Steere et al., 2004; Radolf et al., 2012). LD, the most common vector-borne illness in the U.S. and Europe, is caused by the *Borrelia burgdorferi* sensu lato complex, consisting of 20 species of which 6 cause illness in humans (Rudenko et al., 2011). *B. burgdorferi* sensu stricto (*B. burgdorferi*) causes most infections in the U.S., whereas this species as well as *B. garinii* and *B. afzelii* cause most infections in Europe (Baranton et al., 1992; Canica et al., 1993; Steere et al., 2004). LD *borreliae* are transmitted by *Ixodes* ticks to reservoir animals and humans (Steere et al., 2004). After a tick bite, the bacteria infect the skin at the feeding site, often accompanied with the development of an erythema migrans skin rash (Steere et al., 2004). If left untreated, LD *borreliae* are capable of disseminating to tissues and organs to cause diverse manifestations including arthritis, carditis, and neuroborreliosis (Steere et al., 2004). Human RF infections are transmitted by ticks or lice, resulting in tick-borne relapsing fever (TBRF), or louse-borne relapsing fever (LBRF; Cutler, 2015). At least 10 species of TBRF *borreliae*, including *Borrelia hermsii, Borrelia parkeri*, and *B. duttonii*, are transmitted through bites by various *Ornithodoros* ticks whereas LBRF *B. recurrentis* is solely transmitted by the clothing louse *P. humanus* via crushed lice or feces contacting irritated human skin. Upon transmission, RF *borreliae* cause bacteremia, and alternating febrile/afebrile episodes corresponding with antigenic variation (Cutler, 2015). The spirochetes then disseminate to the central nervous system and may lead to complications in the brain, lungs, kidneys, and spleen (Dworkin et al., 2008; Cutler, 2015).

**Figure 1:**
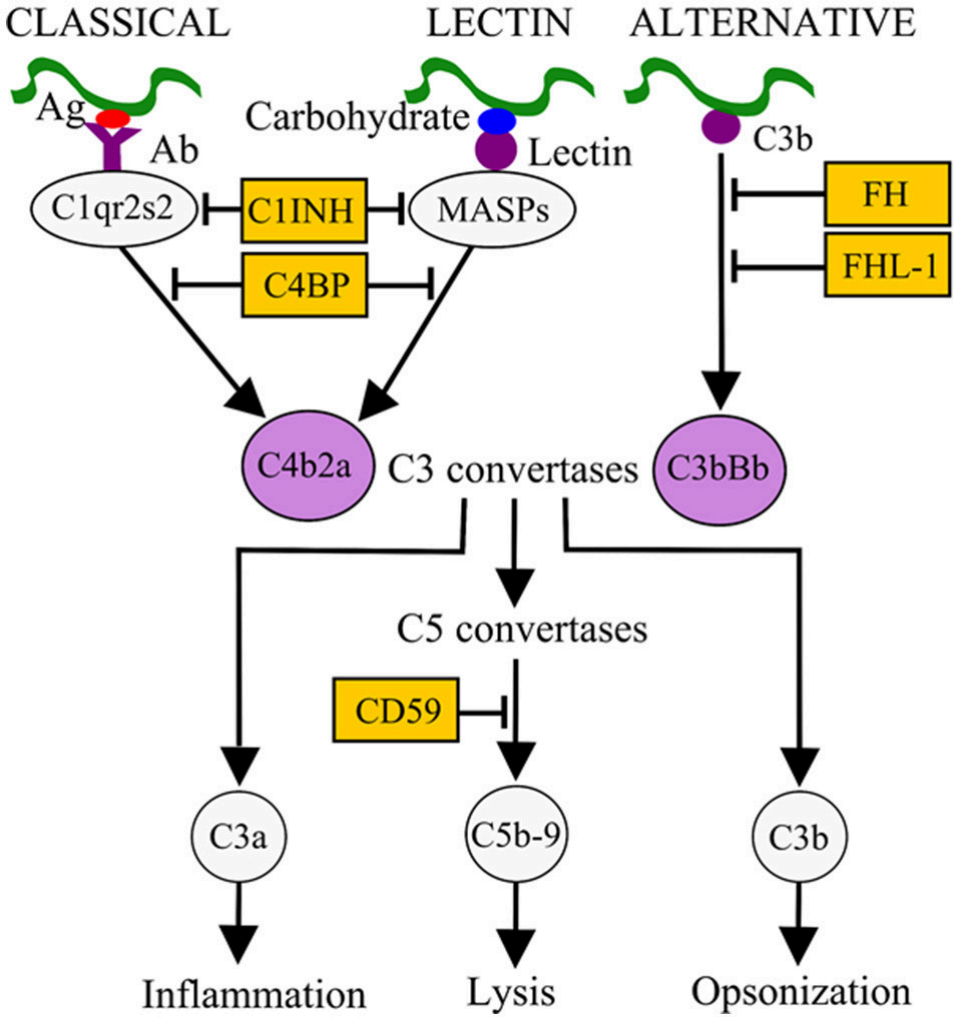
**Activation and control mechanisms of the human complement system**. The classical pathway, initiated by antibody (Ab)-pathogen antigen (Ag) complexes, and the lectin pathway, initiated by lectin-microbial carbohydrate complexes, generate the C3 convertase C4b2a. The alternative pathway, initiated by interaction of C3b with the microbial surface, generates the C3 convertase C3bBb. These C3 convertases, by recruiting other complement components, generate C5 convertases (C4b2a3b and C3bBb3b), which in turn result in the release of pro-inflammatory peptides (C5a), deposition of opsonins (C3b) on the microbial surface to enhance phagocytic clearance, and generation of the membrane attack complex (MAC or C5b-9). Different complement regulators exists to modulate complement activation. For example, C1 inhibitor (C1-INH) binds to C1r/C1s or MASPs and inhibits their proteolytic activity, thus inactivating the classical and lectin pathways. C4BP binds to C4b, factor H (FH), and factor H-like protein 1 (FHL-1) bind to C3b on C3 convertase. These interactions recruit factor I (FI) to inactivate C3b and subsequent activation steps.

Survival in the bloodstream is thought to be essential for LD and RF *borreliae* to cause systemic disease. The complement system is an innate immune defense mechanism in the bloodstream of humans and other vertebrate animals against pathogens (Zipfel and Skerka, 2009). The complement system can be activated via three pathways: classical, lectin, and alternative, all of which result in the formation of C3 convertases (Figure 1). The classical pathway is initiated by the active form of C1 complex (C1qr2s2) binding to antibody-bacterial antigen complexes. The lectin pathway is initiated by binding of lectins [mannan-binding lectin (MBL) or ficolins] to an MBL serine protease (MASP) and microbial carbohydrate. Activation of these pathways leads to the generation of the C3 convertase C4b2a. The alternative pathway is initiated by interaction of C3b with the microbial surface and generates the C3 convertase C3bBb. Both C3 convertases recruit C3b to form C5 convertases, which further promotes the formation of C5b-9 membrane attack complex (MAC) and pathogen lysis. The activation of complement also promotes the release of proinflammatory peptides (C3a and C5a) and deposition of opsonic C3b molecules on the microbial surface to enhance phagocytic clearance (Figure 1). To avoid potential self-damage due to complement activation, vertebrate animals produce a number of diverse complement regulators to negatively regulate the complement system (Figure 1). Examples include C1 inhibitor (C1-INH), which binds to inactive C1rs and/or MASP to block the initiation of the classical and/or lectin pathways. Factor H (FH) and FHL-1 (a truncated form of FH) both bind to and promote the cleavage of C3b via recruiting the protease factor I (FI) to prevent the formation of C3 convertase C3bBb. C4b-binding protein (C4BP) binds to and triggers the degradation of C4b via recruiting FI to inhibit the formation of the C3 convertase C4b2a. Lastly, CD59 binds to C8 and C9 to block the formation of the MAC to avoid lysis of host cells.

Bacterial pathogens, including LD *borreliae*, produce outer surface proteins that bind and recruit complement regulators on the cell surface to inhibit complement activation and prevent killing (Table 1 for references; Kraiczy, 2016). *B. burgdorferi* and *B. garinii* produce the C4BP-binding protein p43, which may recruit C4BP to the bacterial surface to promote C4b degradation and eventually inhibit both classical and lectin pathways. Except *B. bavariensis*, all other serum-resistant LD *borreliae* produce up to five Complement Regulator-Acquiring Surface Proteins (CRASPs): CRASP-1 (CspA), CRASP-2 (CspZ), CRASP-3 (ErpP), CRASP-4 (ErpC), and CRASP-5 (ErpA). CspA and CspZ bind FH (and/or FHL-1). These proteins simultaneously bind C3b and then promote C3b degradation on spirochete surface to downregulate the alternative pathway (Meri et al., 2013). ErpP, ErpC, and ErpA facilitate serum resistance of LD *borreliae* and bind to FH, but the biological significance of these interactions is unclear.

**Table 1:** **LD and RF *borreliae* complement binding proteins and the outcomes of the strategies utilized to demonstrate their functions**.

**Complement binding protein**	**Genospecies**	**Interacting host protein(s)^a^**	**Outcomes of strategies used to determine the function of complement regulators**						**References**
			**Serum resistance**	**Far Western blotting**	**Adsorption assay**	**Hemolytic/Cell-free assay**	**Co-factor assay**	**Deposition assay**	
**LD *borreliae***									
CspA (CRASP-1, BbCRASP-1, BBA68, ZS7, A68, FHBP)	*B. burgdorferi*	C7, C8, C9	+^b^(GOF^c^) (LOF^d^)	ND^e^	ND	+	ND	+	Hallstrom et al., 2013
		FH, FHL-1	+ (GOF) (LOF)	+	+	+	+	+	Kraiczy et al., 2001b, 2004; McDowell et al., 2006; Kenedy et al., 2009; Brooks et al., 2005; Hammerschmidt et al., 2014
	*B. afzelii*	C7, C8, C9	+ (GOF)	ND	ND	+	ND	+	Hallstrom et al., 2013
		FH, FHL-1	+ (GOF)	+	+	+	+	+	Kraiczy et al., 2001a; Hammerschmidt et al., 2014
	*B spielmanii*	C7, C8, C9	+ (GOF)	ND	ND	+	ND	+	Hallstrom et al., 2013
		FH	+ (GOF)	+	+	+	+	+	Seling et al., 2010; Hammerschmidt et al., 2014
CspZ (CRASP-2, BbCRASP-2, BBH06)	*B. burgdorferi*	FH	+ (GOF); ^−^^f^(LOF)	+	+	+	+	+	Kraiczy et al., 2001b; Hartmann et al., 2006; Herzberger et al., 2007; Rogers and Marconi, 2007; Coleman et al., 2008; Siegel et al., 2008
		FHL-1	+ (GOF); − (LOF)	±^g^	+	+	+	+	Kraiczy et al., 2001b; Hartmann et al., 2006; Herzberger et al., 2007; Rogers and Marconi, 2007; Coleman et al., 2008; Siegel et al., 2008
	*B. afzelii*	FH, FHL-1	ND	±	ND	ND	ND	ND	Kraiczy et al., 2001a; Rogers and Marconi, 2007
	*B. spielmanii*	FH, FHL-1	ND	+	ND	ND	ND	ND	Seling et al., 2010
ErpP (CRASP-3, BbCRASP-3, BBN38	*B. burgdorferi*	FH	− (GOF)	+	+	ND	−	−	Kraiczy et al., 2001b, 2003; Stevenson et al., 2002; Kraiczy et al., 2004; Hartmann et al., 2006; Hovis et al., 2006b
	*B. afzelii*	FH	ND	+	ND	ND	+	ND	Kraiczy et al., 2001a
	*B spielmanii*	FH	ND	+	ND	ND	+	ND	Kraiczy et al., 2001a; Seling et al., 2010
ErpC (CRASP-4, BbCRASP-4)	*B. burgdorferi*	FH	− (GOF)	+	+	−	−	−	Kraiczy et al., 2001b; Stevenson et al., 2002; Hartmann et al., 2006; Hovis et al., 2006b; Hammerschmidt et al., 2012
	*B. afzelii*	FH	ND	+	ND	ND	ND	ND	Kraiczy et al., 2001a
ErpA (CRASP-5, BbCRASP-5, ErpI, ErpN, BBP38, BBl39, OspE)	*B. burgdorferi*	FH	− (GOF)	+	+	ND	−	−	Kraiczy et al., 2001b; Stevenson et al., 2002; Alitalo et al., 2005; Hartmann et al., 2006; Hovis et al., 2006b
	*B. afzelii*	FH	ND	+	ND	ND	ND	ND	Kraiczy et al., 2001a
	*B. garinii*	FH	+ (GOF)	+	ND	ND	ND	ND	Kraiczy et al., 2001a
	*B. lusitaniae*	FH	ND	+	ND	ND	ND	ND	Dieterich et al., 2010
BBK32	*B. burgdorferi*	C1r	+ (GOF)	+	ND	+	NA^h^	ND	Garcia et al., 2016
BGA66	*B. bavariensis*	C7, C8, C9	+ (GOF)	+	+	+	+	+	Hammerschmidt et al., 2016
BGA71	*B. bavariensis*	C7, C8, C9	+ (GOF)	+	+	+	+	+	Hammerschmidt et al., 2016
CD59-like protein	*B. burgdorferi*	C9	ND	+	ND	−	ND	ND	Pausa et al., 2003
p43	*B. burgdorferi*	C4BP	ND	+	ND	ND	ND	ND	Pietikainen et al., 2010
	*B. garinii*	C4BP	ND	+	ND	ND	ND	ND	Pietikainen et al., 2010
**RF *borreliae***									
BhCRASP-1 (FhbA, FHBP19, BpcA)	*B. hermsii*	FH, FHL-1	+ (GOF); − (LOF)	+	+	ND	+	ND	McDowell et al., 2003; Hovis et al., 2004, 2006a, 2008; Rossmann et al., 2007; Fine et al., 2014
	*B. parkeri*	FH	+ (GOF)	+	ND	ND	+	ND	McDowell et al., 2003; Schott et al., 2010
CihC (BHA007)	*B.duttonii*	C4BP, C1-INH	ND	±	ND	ND	NC	ND	Grosskinsky et al., 2010; Brenner et al., 2013
	*B. recurrentis*	C4BP, C1-INH	+ (GOF)	+	ND	ND	+	ND	Grosskinsky et al., 2010; Brenner et al., 2013
	*B. hermsii*	C4BP	ND	−	ND	ND	ND	ND	Brenner et al., 2013; Lewis et al., 2014
FHBP28	*B. parkeri*	FH	ND	+	ND	ND	ND	ND	McDowell et al., 2003
HcpA	*B. recurrentis*	FH	+ (GOF)	+	ND	ND	+	ND	Grosskinsky et al., 2009

a *The interacting proteins are from human origin*.

b *+, A positive result shown in the respective strategy*.

c *GOF, Gain of function strains*.

d *LOF, Loss of function strains*.

e *ND, Not determined, which indicates the strategy has not been utilized to study the specific activities*.

f *− A negative result shown in the respective methodology*.

g *±, inconsistent results have been reported*.

h *NA, Not applied because the indicated assay is not applicable to determine the specific activities*.

Similar to LD *borreliae*, RF *borreliae* produce complement regulator-binding proteins on their surface [Table 1 for references; Embers and SpringerLink, (Online service), 2012]. BHA007 in *B. hermsii* and its homolog CihC in both *B. recurrentis* and *B. duttonii* bind C4BP. CihC also binds C1-INH. The association of these proteins with C1-INH and C4BP on the surface of spirochetes prevents the formation of C1 and MASP complexes and induces the cleavage of C4b, respectively, to presumably inhibit the classical and lectin pathways. BhCRASP-1 and FhbA in *B. hermsii*, BpcA in *B. parkeri*, and HcpA in *B. recurrentis* bind FH (and/or FHL-1), which promotes C3b cleavage on bacterial surface and inhibits the alternative pathway.

LD *borreliae* also produce other outer surface proteins that interact with complement components to inhibit the formation of complement complexes and negatively modulate the complement system (Table 1 for references; Kraiczy, 2016). BBK32 of *B. burgdorferi*, known for both fibronectin (Probert and Johnson, 1998) and glycosaminoglycan binding (Fischer et al., 2006), was recently reported as a C1r-binding protein. By binding to the inactive form of C1r, BBK32 blocks the formation of the active C1 complex and inhibits the classical pathway. CspA of *B. burgdorferi, B. afzelii*, and *B. spielmanii*, and BGA66 and BGA71 of *B. bavariensis*, bind C7, C8, and C9. An unknown CD59-like protein of *B. burgdorferi* binds C9. These interactions result in the inhibition of MAC, thereby preventing bacteriolysis.

## Approaches to study mechanisms of serum resistance factors in LD and RF *borreliae in vitro*

### Serum resistance assays

Investigating the role of spirochete proteins in interfering complement pathways allows us to elucidate the mechanisms of bacterial bloodstream survival. Because complement components and regulators are present in the blood, serum resistance assays (also known as bactericidal, growth inhibition, and serum susceptibility assays) are frequently utilized to determine the ability of spirochetes to survive in the serum *in vitro*, which is likely correlated with their ability to survive in the bloodstream *in vivo*. Bacterial survival can be determined by (i) counting viable cells using dark field microscopy, (ii) measuring the color change of the culture media (bacterial growth leads to the acidification of the media, resulting in color change), (iii) staining the DNA of live and dead bacteria, or (iv) plating bacteria on semi-solid agar plates (Table 1 for references). To test the role of a specific protein for serum resistance of LD and RF *borreliae*, spirochetes in the infectious background are genetically engineered to be deficient of these proteins (loss-of-function strains), and these strains are expected to be susceptible to complement-mediated killing (Brooks et al., 2005; Kenedy et al., 2009). However, loss-of-function strains currently can only be generated in *B. burgdorferi*. In addition, any redundant functions provided by other proteins involved in serum resistance in such a strain background may make the defect of a single gene undetectable (Coleman et al., 2008; Fine et al., 2014). Therefore, the alternative strategy is to ectopically produce these factors on the surface of the serum-susceptible spirochetes (gain-of-function strains). Frequently used gain-of-function strains include *B. burgdorferi* strains B313 and B314, and *B. garinii* strain G1. Note, B313 and B314 are non-infectious and only harbor six of the 21 plasmids due to repeated *in vitro* passaging (Sadziene et al., 1993). Gain-of-function strains allow us to study a serum resistance factor without complications from redundant serum resistance proteins.

The concentration of serum used in these assays is important. Although 10–40% serum has been used, only concentrations above 40% effectively eliminate serum-sensitive spirochetes (Breitner-Ruddock et al., 1997; van Dam et al., 1997; Kurtenbach et al., 1998; Kraiczy et al., 2000; Hartmann et al., 2006; Meri et al., 2006; Grosskinsky et al., 2009; Kenedy et al., 2009; van Burgel et al., 2010; Hammerschmidt et al., 2012, 2014; Hallstrom et al., 2013; Garcia et al., 2016). Interestingly, bactericidal activity is not consistently observed by serum from laboratory mouse strains (e.g., C3H/HeN, BALB/c, and C57B/6 strains), likely due to instability of mouse complement *in vitro* (Kurtenbach et al., 1998; Ristow et al., 2012; Caine and Coburn, 2015). The serum from white-footed mouse (*Peromyscus leucopus*), the natural reservoir host of LD spirochetes, invariably displayed ability in serum-sensitive bacterial killing, suggesting the serum from this species may be an alternative for rodent serum resistance assays (Rynkiewicz et al., 2013).

### Far western blotting and serum adsorption assays

To explain the molecular mechanism of serum resistance by LD and RF *borreliae*, Far western blotting (also known as ligand affinity blotting) and adsorption assays have been utilized to determine if complement proteins or regulators bind to the outer surface proteins of spirochetes [Table 1 for the references of specific proteins; Embers and SpringerLink, (Online service), 2012]. In Far western blotting, borrelial proteins from lysed cells are separated on a blot and incubated with either a complement component, regulator, or serum, and then treated with antibodies for detection of the bound complement components or regulators. Reverse ligand blotting, a modified version of Far western blotting, separates serum proteins by size on a blot. The blot is incubated with a purified complement component- or regulator-binding protein and treated with antibodies to detect the complement component- or regulator-binding protein. However, as the binding of these components or regulators to borrelial proteins occurs on the spirochete surface, lysing the cells prior to incubation may change the structure of borrelial proteins and prevent binding. This may explain some inconsistent results when analyzing the complement regulator-binding activity of *borreliae* using this method (Table 1; Hartmann et al., 2006; McDowell et al., 2006; Rogers and Marconi, 2007; Bhide et al., 2009; Grosskinsky et al., 2010; Brenner et al., 2013).

Unlike Far western blotting, serum adsorption assays immobilize whole bacterial cells. After incubating the cells with either complement components, regulators, or serum, bound cells are lysed, separated by SDS-PAGE, and detected by antibodies. This is a more biologically-relevant approach because binding of complement components or regulators occurs under physiological settings on the spirochete surface. Both techniques, however, rely on antibodies for binding detection. As some complement components or regulators (e.g., FH) are polymorphic between animal species (Blom et al., 2004), antibodies against complement components or regulators from one species may not effectively recognize that from another species (McDowell et al., 2006; Rogers and Marconi, 2007), making research in infrequently studied animals inconvenient.

### Hemolytic and cell-free assays

Hemolytic assays have been utilized to quantitatively determine the ability of LD or RF *borreliae* to negatively modulate each complement pathway via complement-component or -regulator-binding proteins. These assays incubate human serum with foreign erythrocytes and borrelial proteins, and measure the level of erythrocyte lysis (Table 1 for references of specific proteins; Dodds and Sim, 1997; Morgan, 2000). These proteins recruit complement components (e.g., C3b, C4b, C7, or C9) by either directly binding to these components or to complement regulators that simultaneously associate with these complement components. This binding reduces the concentration of said complement components in the serum and ultimately inhibits erythrocyte lysis. To maximize hemolysis triggered by the classical pathway or the MAC, erythrocytes are sensitized by pre-incubating with antibodies and the C5b-6 complex, respectively, prior to adding serum. Note, erythrocytes do not need to be incubated with any additional activators prior to adding serum to measure the hemolytic activity induced by the alternative pathway. A lower concentration of serum (1%) can be used to measure the erythrocyte lysis from classical pathways or MAC formation, whereas a higher concentration of serum (above 2.5%) permits detection of hemolysis caused by the alternative pathway (Dodds and Sim, 1997; Morgan, 2000; Hallstrom et al., 2013; Hammerschmidt et al., 2016). Thus, both the serum concentration and the activators used to sensitize erythrocytes are critical to differentiate the pathway-specific hemolysis. In addition, serum deficient in one or more complement components or regulators essential to activation of each pathway can be used to determine which pathways the complement component- or regulator-binding proteins inhibit.

WIESLAB® recently developed a cell-free assay (Wielisa) to quantitatively measure the activation of different complement pathways, which has been used to study spirochete complement component- or regulator-binding proteins (Garcia et al., 2016; Hammerschmidt et al., 2016). Serum incubated with spirochete complement component- or regulator-binding proteins is added to microtiter plates that have been coated with immobilized immunoglobulin (classical pathway), mannan (lectin pathway), or lipopolysaccharides (alternative pathway). The ability of these bacterial proteins to inhibit complement activation is determined by detecting the level of MAC formed on the surface of microtiter plates.

### Cofactor assays

Cofactor assays determine if complement regulators bound by spirochete proteins facilitate the cleavage of the target complement components [Table 1 for references of specific proteins; Embers and SpringerLink, (Online service), 2012]. For example, following the binding of complement regulators to the immobilized protein or spirochete surface, the ability of FH (or FHL-1) to promote C3b degradation in the presence of FI can be detected by identifying cleaved C3b using Western blotting. The ability of spirochete C4BP-binding protein to promote C4b degradation by binding to C4BP and FI can also be performed in a similar fashion. Although the concentrations of the complement regulator-binding proteins used in this assay are generally higher than what is likely physiologically relevant, this technique allows us to demonstrate a molecular mechanism of these proteins in inactivating complement system by binding to respective regulators.

### Deposition assays

Complement complexes form on the surface of spirochetes during complement activation (Table 1 for references of specific proteins). Therefore, detecting C3b (a component of C3 and C5 convertases), and C6 and C5b-9 (the components of MAC) allows us to measure the level of complement activation on the surface of LD or RF *borreliae*. Deposition assays utilize immunofluorescence staining or ELISA to measure the levels of the aforementioned complement components bound on the bacterial surface after spirochetes strains are incubated with serum. LD and RF *borreliae* that bind complement components or regulators from serum should have reduced or no deposition of C3b, C6, and C5b-9. Note, serum concentrations used range from 10 to 25% because serum concentrations >40% eliminate *Borrelia*, which prevents observation of complement deposition (Kurtenbach et al., 1998; Kenedy et al., 2009; Hammerschmidt et al., 2014).

## Approaches to study bloodstream survival provided by the factors in LD or RF *borreliae in vivo*

In the natural transmission of LD or RF *borreliae* from ticks to vertebrate animals, the spirochetes first colonize the skin at the tick feeding site prior to disseminating into the bloodstream and migrating into the surrounding tissues (Radolf et al., 2012; Coburn et al., 2013). In traditional models, mice are inoculated subcutaneously or intradermally, or by bite from a tick infected with LD or RF *borreliae* to study the contribution of spirochete factors during infection (Barthold et al., 1990; Simon et al., 1991). However, since a failure at either initial skin colonization or bloodstream survival would lead to low or undetectable bacterial burdens in the animal, it can be difficult to distinguish the roles of spirochete factors in promoting survival within the mammalian host using traditional models.

A short-term murine model has recently been developed using the LD spirochete *B. burgdorferi* to investigate the roles of bacterial outer surface proteins in mammalian bloodstream survival (Caine and Coburn, 2015). This model intravenously inoculates mice with a high number of spirochetes for up to 1 h. The ability of the spirochetes to survive in the bloodstream can be detected by measuring bacteremia (Caine and Coburn, 2015). Intravenous inoculation bypasses the initial step of skin colonization allowing the study of non-infectious mutant spirochetes. Therefore, this strategy teases apart the contributions of *Borrelia* factors with multiple functions in bloodstream survival, protein adhesion, and tissue attachment. For example, *B. burgdorferi* outer surface protein BBK32 contributes to colonization of the inoculation site of skin (Seshu et al., 2006; Hyde et al., 2011; Lin et al., 2015). Whether this protein contributes to mammalian bloodstream survival during Lyme infection is difficult to assess by subcutaneous needle or tick infection. Using short-term intravenous inoculation in a murine model, BBK32 ectopically-produced on a non-infectious, serum-sensitive *B. burgdorferi* strain promotes spirochete survival in the bloodstream (Caine and Coburn, 2015). This strategy has also been applied to identify the contribution of other *B. burgdorferi* factors in promoting bloodstream survival (Caine and Coburn, 2015). As RF *borreliae* are also blood-borne pathogens that disseminate into host tissues, this short-term model could be employed to further characterize serum resistance and disease progression in RF *borreliae*. Though some complement components or regulator are polymorphic among vertebrate animals (Lu et al., 2008), which raises a concern that the *in vivo* murine models may not be relevant to humans, recent developed humanized mouse strains may be utilized as a solution of this issue (Beernink et al., 2012).

## Conclusion

Bloodstream survival of LD or RF *borreliae* is thought to be essential for spirochetes to survive in humans, and ultimately cause LD or RF disease manifestations. Serum resistance, adsorption, hemolytic, cofactor, and deposition assays, as well as a recently established short term intravenous inoculation murine model are all used to elucidate the mechanism of LD and RF *borreliae* evasion of the complement system and survival in the bloodstream. The data reviewed here are mainly on borrelial interactions with humans, but these assays can also be applied to the interactions with other vertebrate hosts, which will elucidate the role of the borrelial complement evasion in the enzootic cycle. Understanding these mechanisms in both humans and other vertebrate hosts will aid in the development of novel therapeutic strategies and disease prevention by targeting these complement component- or regulator-binding proteins to ultimately improve human health.

## Author contributions

AM, PK, and YL wrote the manuscript; and AM and YL prepared the figure and table.

### Conflict of interest statement

The authors declare that the research was conducted in the absence of any commercial or financial relationships that could be construed as a potential conflict of interest.

## Abbreviations

Ab: Antibody
Ag: Antigen
B313: *B. burgdorferi* strain B313
B314: *B. burgdorferi* strain B314
C4BP: C4b-binding protein
CRASP: Complement Regulator-Acquiring Surface Protein
FH: Factor H
FHL-1: Factor H-like protein 1
FI: Factor I
G1: *B. garinii* strain G1
LBRF: louse-borne relapsing fever
LD: Lyme disease
MAC: membrane attack complex
MASP: Mannan-binding lectin serine protease
MBL: Mannan-binding lectin
RF: relapsing fever
TBRF: tick-borne relapsing fever.
